# Lymphoepithelial Carcinoma of the Sublingual Gland: A Case Report

**DOI:** 10.7759/cureus.54305

**Published:** 2024-02-16

**Authors:** Swathi Pavuluri, Alison Caceres, Emily Kwon, Davis Chacko, Imraan Jan, Sung Kim

**Affiliations:** 1 Radiation Oncology, Rutgers University New Jersey Medical School, Newark, USA; 2 Pathology, Rutgers University New Jersey Medical School, Newark, USA; 3 Radiation Oncology, Rutgers Robert Wood Johnson Medical School, New Brunswick, USA

**Keywords:** rare head and neck, salivary gland carcinoma, case report, epstein- barr virus, sublingual gland, lymphoepithelial carcinoma

## Abstract

Lymphoepithelial carcinoma (LEC) of the salivary gland is a rare squamous cell carcinoma. LEC commonly presents in the parotid and submandibular glands and rarely in the sublingual gland. While salivary gland LEC has a predilection for Inuit-Yupik and Chinese populations, few cases have been reported in the Hispanic population and none for sublingual glands in the English language literature. Here, we present the seventh case report in the English language literature for sublingual LEC and the first case observed in a Hispanic patient.

## Introduction

Lymphoepithelial carcinoma (LEC) is an undifferentiated carcinoma with lymphoid stroma. Hilderman et al. (1962) first described LEC of the salivary gland, where the tumor originated in the parotid gland and was defined as the “malignant counterpart of benign lymphoepithelial lesions” [1]. It is endemic to the Inuit-Yupik and Chinese populations in the Arctic and Asian regions and is most strongly associated with the Epstein-Barr Virus (EBV) [2]. Patients affected by LEC span a wide age range (10-86 years), with the mean and median age of cases occurring in the fourth decade [3]. 

LEC typically presents in the nasopharynx and is rarely observed in the oropharynx [4]. A rare tumor of the salivary gland, LEC accounts for less than 1% of all malignant salivary gland tumors [5]. Of the salivary gland cases, LEC develops most frequently in the parotid gland (82%) and the submandibular gland. LEC is not typically observed in the sublingual gland [4]. Limited literature exists, therefore, describing LEC of the salivary gland. LEC in the sublingual gland has been reported only six times in the existing English-language literature [2,5-9]. In addition, to the best of the authors’ knowledge, this is the first reported case of a Hispanic individual with sublingual LEC. Salivary LECs are not commonly observed in Hispanic populations; to date, only three parotid gland cases have been reported in Hispanic individuals [10-11]. Here, we present the seventh case report in the English literature for sublingual LEC and the first case report of this neoplasm in a Hispanic patient.

## Case presentation

A 43-year-old Hispanic woman presented for the evaluation of an enlarged right sublingual gland. The patient indicated that she had the mass for five years and that it had been stable in size until January 2021, when it began noticeably growing. She denied any recent upper respiratory infection, dysphagia, change in voice, and unintended weight loss. The patient had no significant past medical history and no reported family history of malignancy. She was only on naproxen at the time, which had been prescribed by her primary care provider for mild joint pain. She did not smoke, drink alcohol, or use illicit drugs and had no known allergies. On physical examination, a right submandibular 1-cm mass was palpable without tenderness, nodules, or cervical lymphadenopathy. Oral and buccal mucosa was pink and moist, tonsils were +1 and non-erythematous without exudates, and no floor-of-mouth masses or lesions were seen. Facial nerve function was grossly preserved. The nasopharynx was free of disease as seen on flexible laryngoscopy later in November 2021.

**Figure 1:**
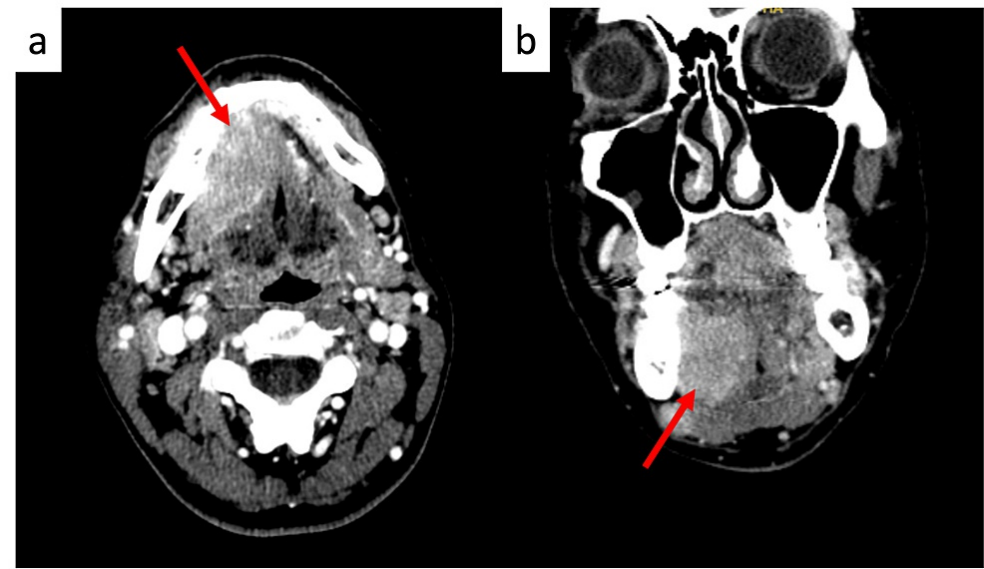
Axial (a) and coronal (b) CT head and neck images with contrast A 3.6 x 2.1 x 2.6 cm enhancing mass centered in the right aspect of the floor of the mouth (red arrows)

CT imaging demonstrated a 3.6 x 2.1 x 2.6 cm enhancing mass centered in the right aspect of the floor of the mouth (Figure 1). The mass exerted a mass effect on the right geniohyoid muscle and appeared inseparable from the mylohyoid muscle below. The mass bordered the lingual surface of the right mandible without causing visible erosion or destruction of the adjacent bony cortex. A 2.1 x 1.8 x 2.0 cm lymph node was also observed in the right level Ib nodal region, which was concerning for metastatic focus and exerted a mass effect on the adjacent submandibular gland.

**Figure 2:**
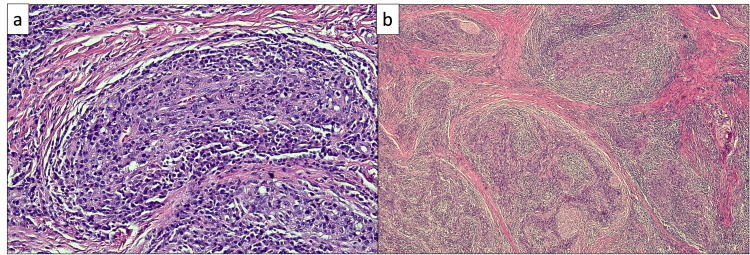
Nests of malignant epithelial cells characteristic of LEC a: Hematoxylin and eosin (H&E), 40x. b: H&E, 5x LEC: lymphoepithelial carcinoma

**Figure 3:**
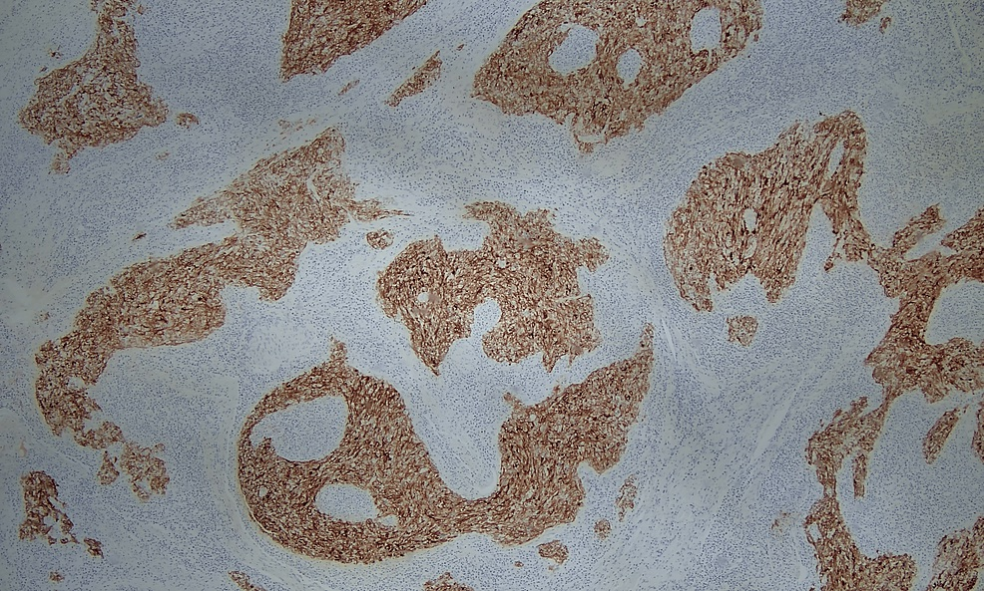
A cytokeratin AE1/AE3 shows diffuse and intense membranous and cytoplasmic positivity (IHC, 10x) IHC: immunohistochemistry

**Figure 4:**
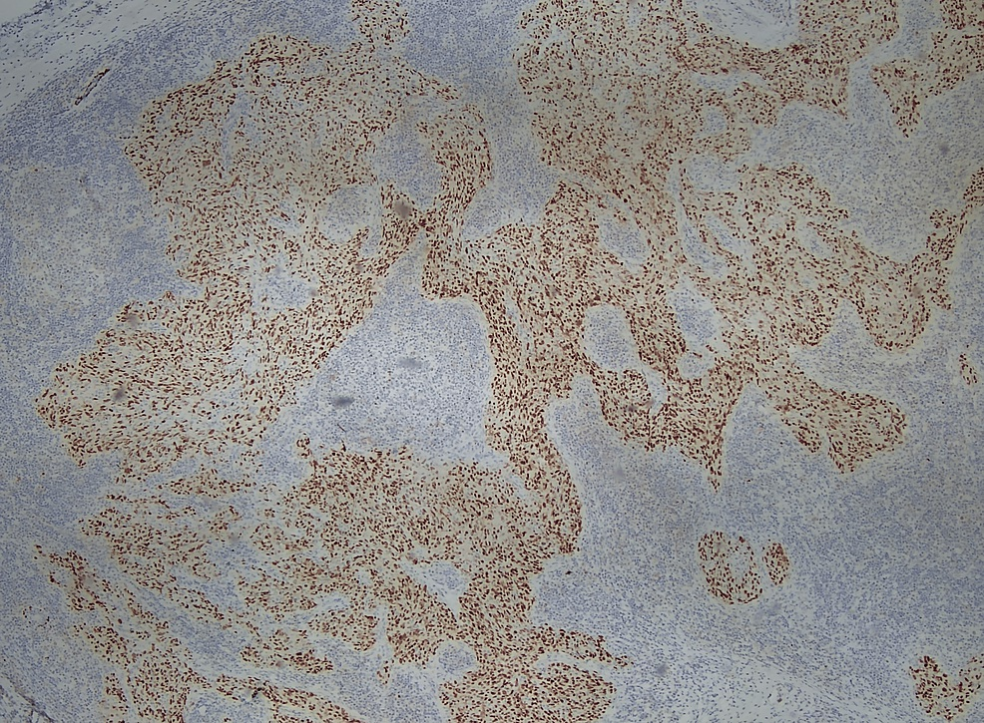
A p63 stain shows diffuse and intense nuclear positivity (IHC, 10x) IHC: immunohistochemistry

**Figure 5:**
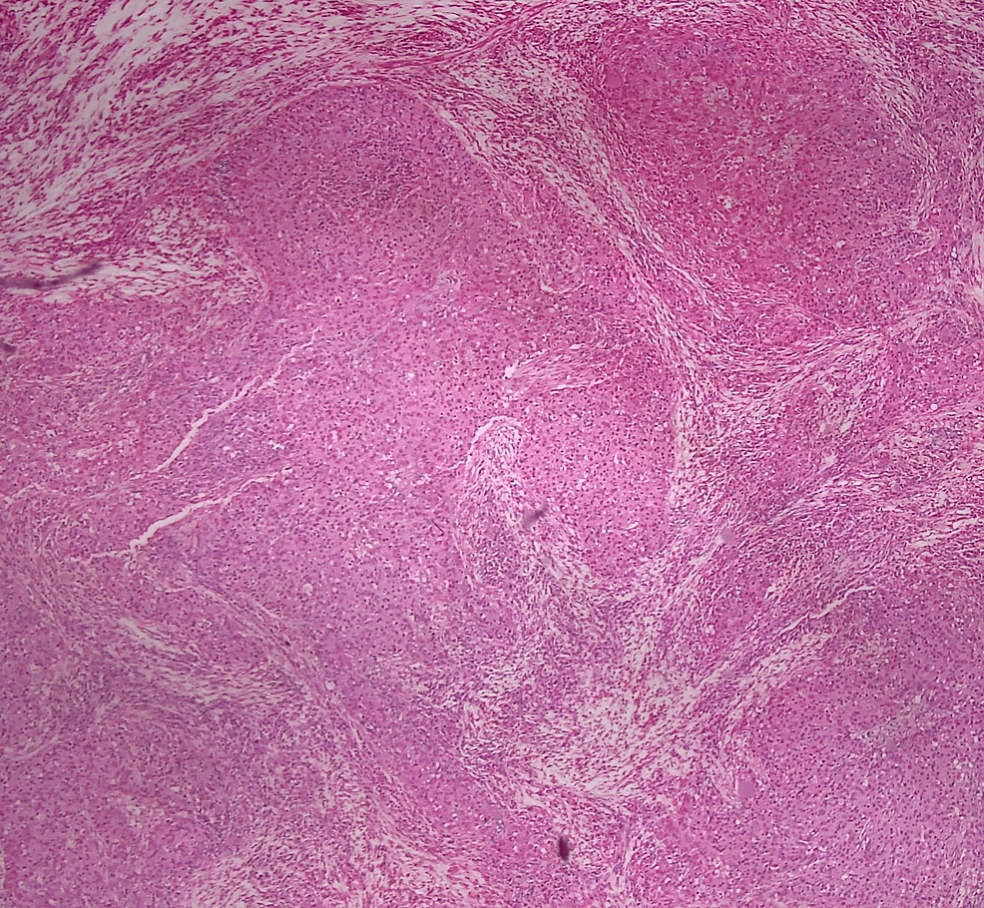
Epstein-Barr virus-associated LEC (EBER ISH, 20x) LEC: lymphoepithelial carcinoma

**Figure 6:**
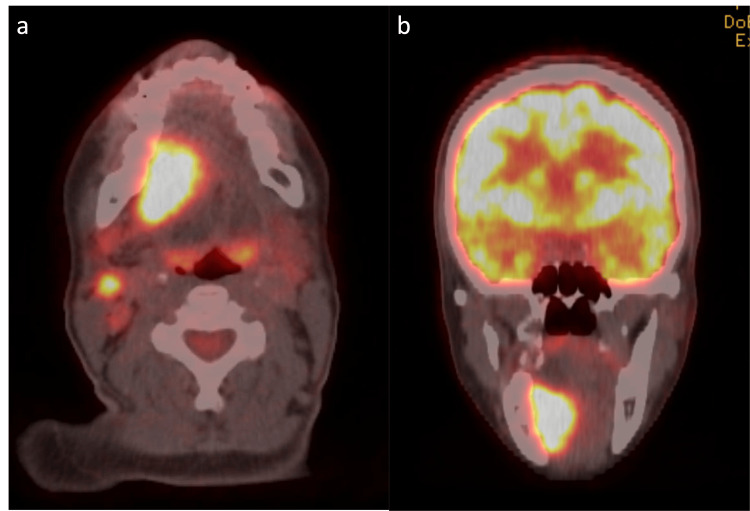
Axial (a) and coronal (b) PET-CT scans showing hypermetabolic activity in the right floor of the mouth and a metastatic lymph node consistent with biopsy-proven malignancy PET-CT: positron emission tomography-computed tomography

Upon a subsequent visit to review the CT scan one month later, the patient reported the emergence of symptoms of numbness in the right side of the tongue while eating and intermittent and self-resolving 4/10 pain in the right side of the neck at night. She also noted that the mass had been growing, which was confirmed on physical examination with the observation of a 2-cm right submandibular mass on palpation. No other symptoms or physical findings were reported. Fine-needle aspiration biopsy was conducted of the neck lymph node on level Ib. Histologically, there existed nests of poorly differentiated epithelial cells within a background of lymphoid infiltrates. These neoplastic cells exhibit a high nuclear: cytoplasmic ratio, along with pleomorphism, irregular nuclei, and prominent nucleoli (Figure 2). Immunohistochemistry (IHC) revealed positive staining for cytokeratin AE1/3, CK 5/6, and P63 (Figures 3-4). Additionally, in situ hybridization for EBV is positive with diffuse staining (Figure 5). These histological and immunohistochemistry studies support the diagnosis of LEC. Laryngoscopy displayed vague slight fullness in the right base of the tongue without ulceration, lesions, or masses elsewhere. The nasopharynx was clear. The PET-CT scan displayed large areas of uptake in the oral cavity tongue on the right side with possible right-sided neck metastasis (Figure 6).

The patient was treated surgically with composite resection of the right floor of the mouth/ventral tongue, right supraomohyoid neck dissection (levels I-III), tracheostomy tube placement (6DCT), radial forearm free flap from left, and split-thickness skin graft from left thigh. The patient tolerated the surgery well. Definitive pathological staging was Stage III T3N1M0. Adjuvant radiation therapy was administered bilaterally using intensity-modulated radiation therapy with 6 MV photons to two different dose levels (60 Gy and 54 Gy in 2 Gy per fraction; Gy = Gray). The radiation was administered to the full tongue, floor of the mouth, and nodal areas Ib, II, III, and IV. The patient tolerated treatment well, with some usual radiation side effects, such as mucositis, skin reaction, dry mouth, and change in taste.

A positron emission tomography (PET) scan conducted three months following the completion of adjuvant radiation therapy was significant for the presence of a 1.5 cm soft tissue nodule at the midline anterior inferior aspect of the right oral cavity/tongue, demonstrating a mild increase in fluorodeoxyglucose (FDG) uptake with a maximum standardized uptake value (SUV) of 2.14. These findings were inconclusive for post-treatment changes versus tumor recurrence. Repeat imaging eight months post-treatment demonstrated the absence of the previous focal uptake on the anterior aspect of the oral cavity/tongue; however, a new focal uptake was present at the mid-anterior region of the tongue in the post-surgical bed, prompting a biopsy that was negative for disease recurrence.

## Discussion

LEC of the salivary gland is a rare poorly differentiated squamous cell carcinoma first described by Hilderman et al. in 1962 [1,9]. Histologically, it appears as islands of atypical epithelioid cells with pale cytoplasm, large vesicular nuclei, and prominent nucleoli, surrounded by a benign lymphoplasmacytic background [12-14].

A review article of 438 LEC in salivary gland cases in 2021 reported an equal incidence of LEC in men and women. Furthermore, 76% of LEC tumors were located in the parotid gland, 17% were located in the submandibular glands, and only 0.4% of tumors were located in the sublingual glands. The ages of those affected were between 10 and 86 years of age, with the mean being 47.1 years old. Out of 438 reported cases, most patients were of Asian descent (73%) and Eskimo descent (11%) [6-8,12]. There were no reports of Hispanic patients in these cases. However, in 1995, Kountakis et al. reported two cases of Hispanic women with right parotid masses, EBV negative, that were both in the preauricular area without involvement of the nasopharynx. In both cases, there was metastasis to lymph nodes in the jugular area. Both patients were treated with parotidectomy and localized radiation (50-60 Gy) to the parotid gland and the neck (25-50 Gy in fractions) [10]. No cases of LEC in the sublingual gland have been reported to date in Hispanic women. 

LEC is an unencapsulated tumor with a strong tendency for metastasis. Over 40% of patients have metastases to the cervical lymph nodes at initial presentation, 20% develop local recurrence or spread to lymph nodes, and 20% develop distant metastases within 3 years of treatment [9,15,16]. Since as many as 90% of neoplasms of the sublingual gland are malignant, masses of the sublingual gland should be considered malignant unless otherwise proven to be benign [6,17]. Imaging and fine-needle aspiration biopsy are the benchmarks for the workup of sublingual lesions. These techniques are also used for standard metastatic workup, given the prevalence of metastases with LECs.

Definitive diagnosis of LEC is based on histopathological findings [2,18]. The LEC differential diagnosis includes benign lymphoepithelial lesion, malignant lymphoma, and metastatic undifferentiated carcinoma. LEC can be differentiated from other tumors through histological examination and immunostaining. However, metastatic undifferentiated nasopharyngeal carcinoma may be morphologically indistinguishable through such analyses [9]. Furthermore, the salivary glands lie adjacent to lymphatic drainage from metastatic undifferentiated nasopharyngeal carcinoma, making it difficult to make the final diagnosis [2,18]. Ruling out a metastatic cause is essential to ensure that a primary tumor receives sufficient treatment [9]. This is accomplished through careful examination of the upper respiratory and digestive systems as well as a biopsy of any suspicious tissue identified in the nasopharynx [9,19].

Treatment options for LECs consist of surgical excision, radiation therapy, and chemotherapy [6,20]. However, the utility of chemotherapy has yet to be validated [2,6,21]. The standard consensus for the treatment of LEC of the salivary gland involves surgical excision and adjuvant radiation therapy, typically around 60 Gy, given the radiosensitivity of LECs [2,6,18,22,23]. The five-year survival rates for this treatment range from 50% to 90%, but the paucity of reported cases of LEC of the sublingual gland continues to serve as a barrier to conclusive statistical outcomes of treatments in this population, particularly given the limited diversity of patient demographics reported in the literature thus far [2].

## Conclusions

Lymphoepithelial carcinoma (LEC) of the salivary gland is a rare squamous cell carcinoma, with few cases documented in the literature and none yet reported in Hispanic populations. Here, we described the case of a young Hispanic woman who received extensive treatment for LEC of the sublingual gland.
